# Molecular Rotors in a Metal–Organic Framework: Muons on a Hyper-Fast Carousel

**DOI:** 10.1021/acs.nanolett.0c03140

**Published:** 2020-09-01

**Authors:** Giacomo Prando, Jacopo Perego, Mattia Negroni, Mauro Riccò, Silvia Bracco, Angiolina Comotti, Piero Sozzani, Pietro Carretta

**Affiliations:** † Department of Physics, University of Pavia, I-27100 Pavia, Italy; ‡ Department of Materials Science, University of Milano Bicocca, I-20125 Milano, Italy; § Department of Mathematical, Physical and Information Sciences, University of Parma, I-43124 Parma, Italy

**Keywords:** Muon-spin spectroscopy, Molecular rotors, Metal−organic frameworks

## Abstract

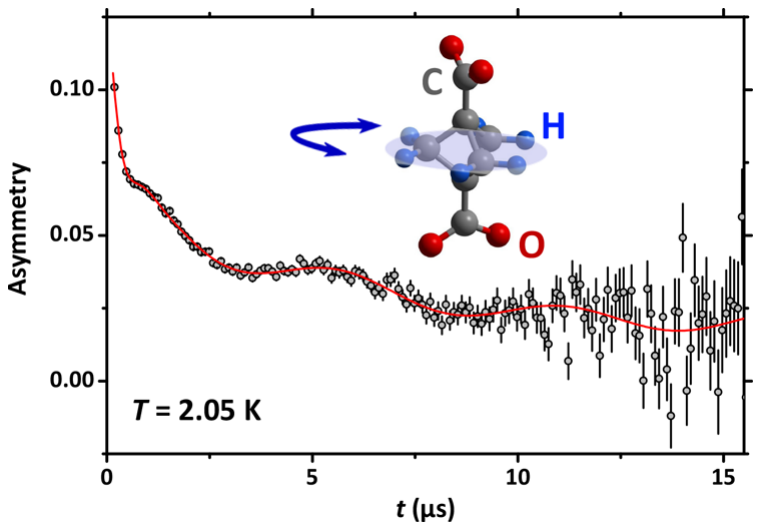

Using muon-spin spectroscopy, we study the exceptional dynamical properties of rotating molecular struts engineered within a Zn-based metal–organic framework at cryogenic temperatures, where the internal motions of almost any other organic substance are quenched. Muon-spin spectroscopy is particularly suited for this aim, as the experimental evidence suggests several implantation sites for the muons, among which at least one directly onto the rotating moiety. The dynamics of the molecular rotors are characterized by the exceptionally low activation energy *E*_A_ ∼ 30 cal mol^–1^. At the same time, we evidence a highly unusual temperature dependence of the dipolar interaction of muons with nuclear magnetic moments on the rotors, suggesting a complex influence of the rotations on the muon implantation and diffusion.

Research on molecular machines, rotors, and switches is a very active field lying at the boundary between chemistry, physics, and materials science whose ultimate aim is to control the mechanical motion of nanoscopic objects in order to implement useful tasks at the nanoscale.^1−8^ The realization of rotational movements for molecules along controlled axes is of significant interest for most applications in this respect.^9,10^ In particular, the design and synthesis of materials endowed with fast dynamics made the prospects for several applications, such as selective sensors, gas sorption and release-on-command, and switchable ferroelectrics, much more concrete.^11,12^

For this aim, the recent use of porous materials as a new platform has extended the perspectives of fast molecular rotors to the crystalline solid state.^12−16^ Within this context, researchers have devoted particular interest to metal–organic frameworks (MOFs), which are engineered by self-assembly of rotor-containing ligands and integrate both stator and rotary elements in the same crystal structure. The highly porous nature of MOFs retains the free volume needed for the rotary motion to occur; at the same time, the accurate design of the surrounding crystal architecture and the low torsional energy profile about pivotal bonds are necessary to push the motional behavior to its limits. By means of these approaches, ultrafast dynamics comparable to liquid matter was demonstrated recently at cryogenic temperatures with characteristic activation energies markedly smaller than the thermal energies at ambient conditions.^17−19^

The in-depth knowledge of dynamical processes in solids is challenging, especially in the presence of fast motional processes. Positive muons are very convenient tools for this aim, since they act as highly sensitive local probes of microscopic fluctuating magnetic fields.^20−25^ Thus, muon-spin spectroscopy (μSR) has been a valuable tool to reveal molecular reorientation in liquid-crystalline materials and molecular dynamics in general.^26−31^ In this work, we report on the first μSR study of the unique dynamical properties of molecular rotors engineered in MOFs, focusing on a Zn-based MOF containing bicyclo-[1.1.1]-pentane-1,3-dicarboxylate struts.^19^ The cubic structure of this compound (Figure 1a) contains nodes made by four Zn atoms with tetrahedral geometry coordinated with six bicyclopentane-dicarboxylic acid ligands. The nodes act as the stator for the rotary motion of the bicyclo-[1.1.1]-pentane rotor. Geometrical frustration arises from the *C*_3_ symmetry of the rotor conflicting with the crossed *C*_4_ arrangement of the two neighboring carboxylate groups (Figures 1b and 1c). This delicate balance of different symmetries favors the generation of very shallow energy minima associated with the orientation of the bicyclo-[1.1.1]-pentane unit whose rotary motion, as a result, is characterized by a virtually complete compensation of the energy barrier. Remarkably, the dynamics is preserved down to cryogenic temperatures where almost any organic substance is solid and internal motions are quenched, with the exception being proton exchange and small moieties, such as amine and methyl groups. Details on the synthesis and characterization of the described MOF are reported in the Supporting Information (Figures SI1, SI2, and SI3).

**Figure 1:**
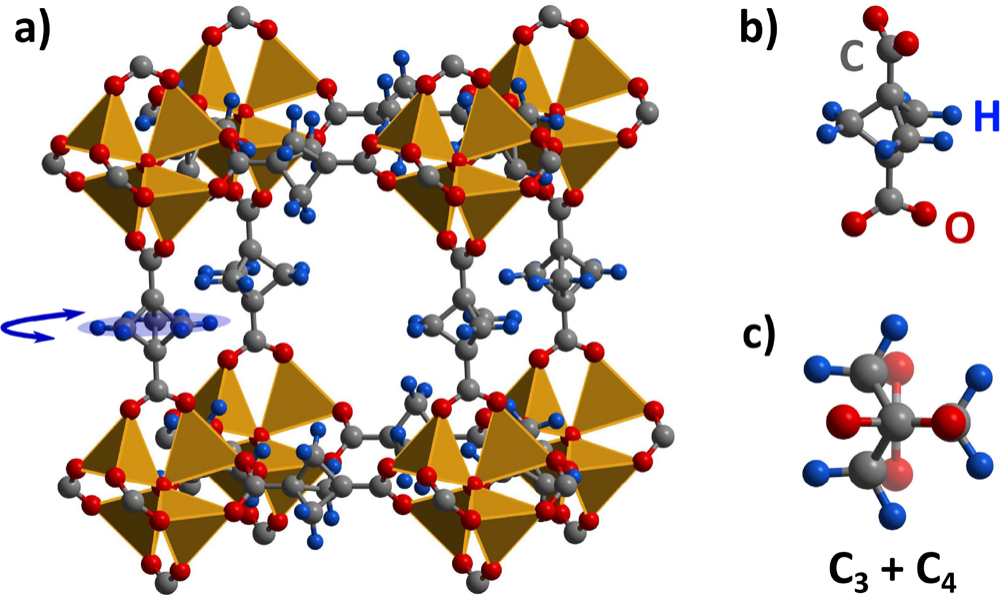
(a) Cubic crystalline structure of the investigated MOF showing the rotating bicycle unit suspended within the Zn-based stators. The bicyclo-[1.1.1]-pentane-1,3-dicarboxylate moieity is also viewed (b) parallel and (c) perpendicular to the rotation axis.

Critical aspects of μSR are the determination of the muon implantation crystallographic sites and the possible influence of the implanted muons on the properties of the investigated materials. For the measurements we are currently reporting on, we have evidence of several inequivalent implantation sites. One site is of particular interest as muons implanted therein are sensitive to the rotational dynamics of the rotor as a whole, evidencing a value *E*_A_ ∼ 30 cal mol^–1^ for the characteristic activation energy for the rotations, i.e., the rotors’ mobility. Although higher than the NMR estimate, likely because of the perturbation induced by the muons on the motions, this exceptionally low value is a benchmark for the easiness of rotation of the studied molecular rotors. On the other hand, the signal from those muons implanted in other site(s) evidences well-defined coherent oscillations arising from the dipolar interaction with the nuclear magnetic moments of hydrogen atoms. This unambiguously locates these latter implantation site(s) directly onto the rotating moiety, where hydrogen atoms are only present. The unprecedented evolution of the oscillating signal upon increasing temperature demonstrates that the implantation of muons onto the rotors is strongly affected by the onset of the rotary dynamics, which may favor muons’ diffusion in turn.

As discussed in the Supporting Information, the main quantity of interest in a μSR experiment is the so-called spatial asymmetry in the muons’ radioactive decays. It can be shown that this quantity is directly proportional to the spin autocorrelation function for the muon, and accordingly, a time-resolved measurement of the decay asymmetry is equivalent to measuring the time-evolution of the spin depolarization of the muons subject to the local static and dynamical magnetic fields probed in the investigated material. We report representative results for the muon depolarization under conditions of zero external magnetic field (ZF) in Figure 2, evidencing a strong dependence of the experimental results on temperature. The value of the full initial asymmetry (∼0.09–0.12) is quite low compared to the maximum instrumental value (∼0.25) regardless of the temperature value. We distinguish three different regimes. In the high-temperature range (HT, *T* ≳ 13 K), the ZF asymmetry function is reproducible as the sum of two decaying contributions with markedly different time constants (∼6 μs^–1^ and ∼0.05 μs^–1^, respectively). Upon cooling the system, in the intermediate-temperature range (IT, 8 ≲ *T* ≲ 13 K), the initial exponentially decaying component evolves progressively in a well-defined coherent oscillation. Suddenly, below the temperature value *T* ≃ 8 K (low-temperature range, LT), the amplitude of the oscillating fraction gets unevenly redistributed between two cosine-like signals with different frequencies.

**Figure 2:**
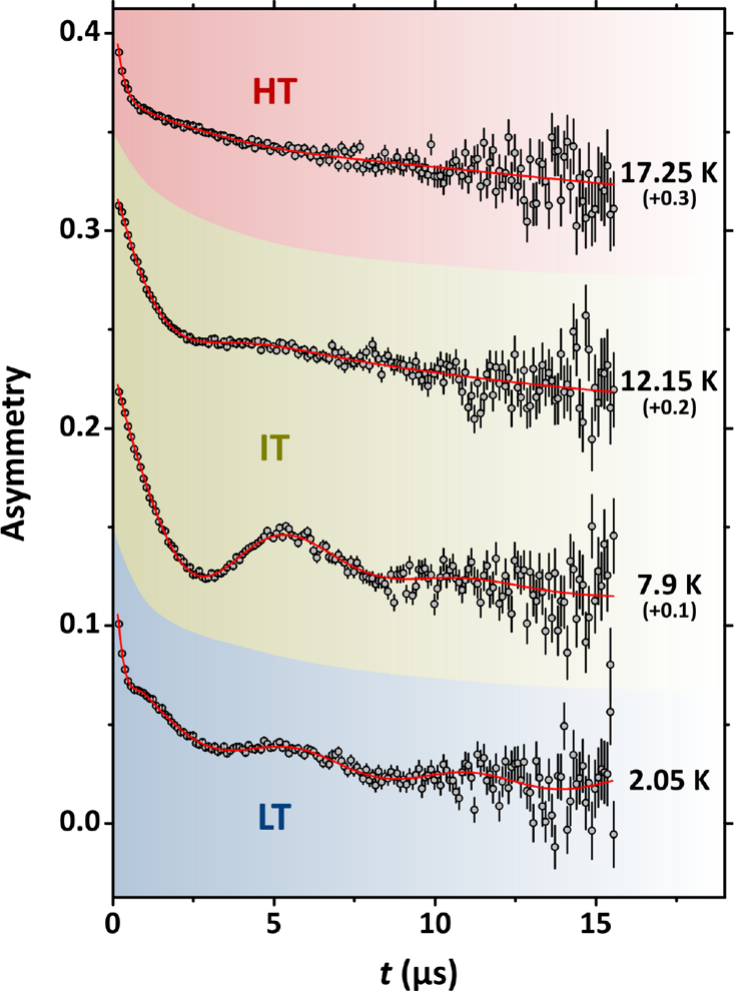
Representative results of ZF-μSR at different temperature values in the low- (LT), intermediate- (IT), and high-temperature (HT) regions. The curves have been shifted vertically by the amounts in the parentheses for the aim of clarity. The continuous lines are best-fitting functions according to eq 1 for the data at 7.9, 12.15, and 17.25 K. For the measurement at 2.05 K, the continuous line is a best-fitting function according to the H−μ model discussed in the Supporting Information (see eq SI11).

Based on the qualitative arguments above, we fitted the following function
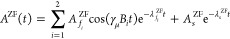
1to the experimental data in ZF conditions. Here, *A*^ZF^ is the total asymmetry of the muons’ decays as a function of time *t*, split into different components with amplitudes *A*_*f*_*i*_,*s*_^ZF^. In general, the two *f* components (labeled by the index *i*) are exponentially damped cosine functions, where the angular frequency is written as the magnetic field at the muon site *B*_*i*_ times the muon gyromagnetic ratio γ_μ_ = 2π × 13.554 rad ms^–1^ G^–1^ and where λ_*f*_*i*__^ZF^ are the associated damping rates. Finally, the long-tail relaxing component is damped exponentially with rate λ_*s*_^ZF^. In order to match the experimental findings described above, we fix *B*_1_ = 0 in the HT region and *A*_*f*_2__^ZF^ = 0 in both the IT and HT regimes. We obtain good fitting results (statistical χ^2^ < 1.1) throughout the whole temperature range.

In the following we will focus also on the effect of external magnetic fields *H*^LF^ parallel to the muons’ spin at the moment of implantation, the so-called longitudinal field (LF) geometry. We report representative experimental curves in LF geometry in the insets to Figure 3 and Figure 4. Under the effect of a longitudinal field, we observe that the depolarization can be described as the sum of two exponentially decaying functions with markedly different time constants; however, as thoroughly discussed later on in the text, only the long-tail component is observed in the LT regime. To reproduce these observations, we refer to the following expression:
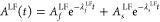
2as best-fitting function for the depolarization curves at all temperatures, provided that *A*_*f*_^LF^ = 0 within the LT region. The meaning of symbols in eq 2 can be derived straightforwardly based on the discussion of eq 1.

**Figure 3:**
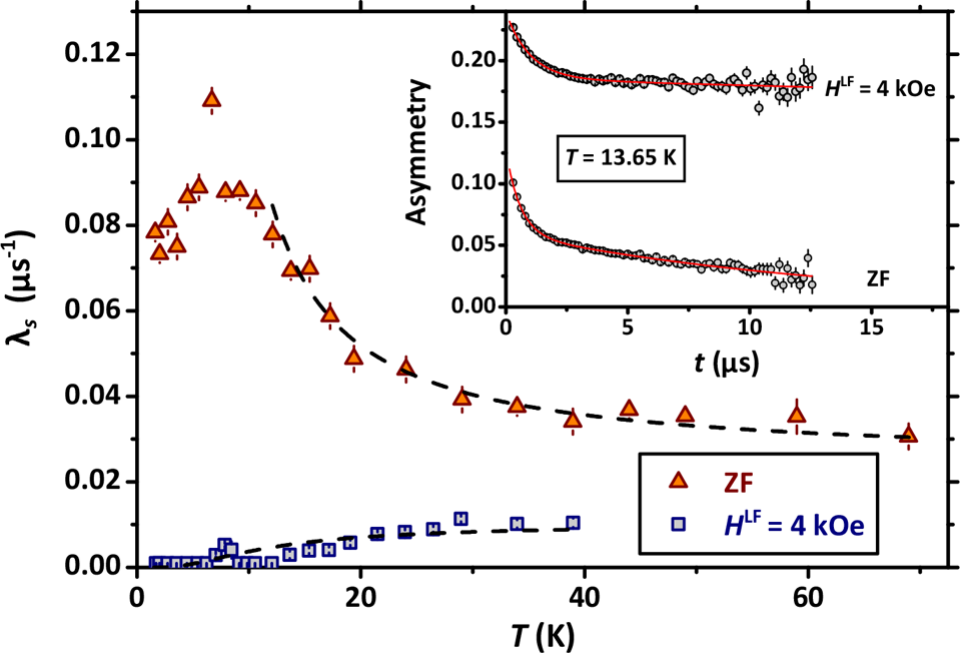
Main panel: temperature dependence of the relaxation rate λ_*s*_ under ZF conditions and for *H*^LF^ = 4 kOe. The dashed curves are the results of a simultaneous best-fitting procedure to both ZF and LF data based on eqs 3 and 4. Inset: representative depolarization curves at *T* = 13.65 K under conditions of ZF and LF (*H*^LF^ = 4 kOe). The continuous lines are best-fitting curves based on eq 1 and eq 2, respectively.

**Figure 4:**
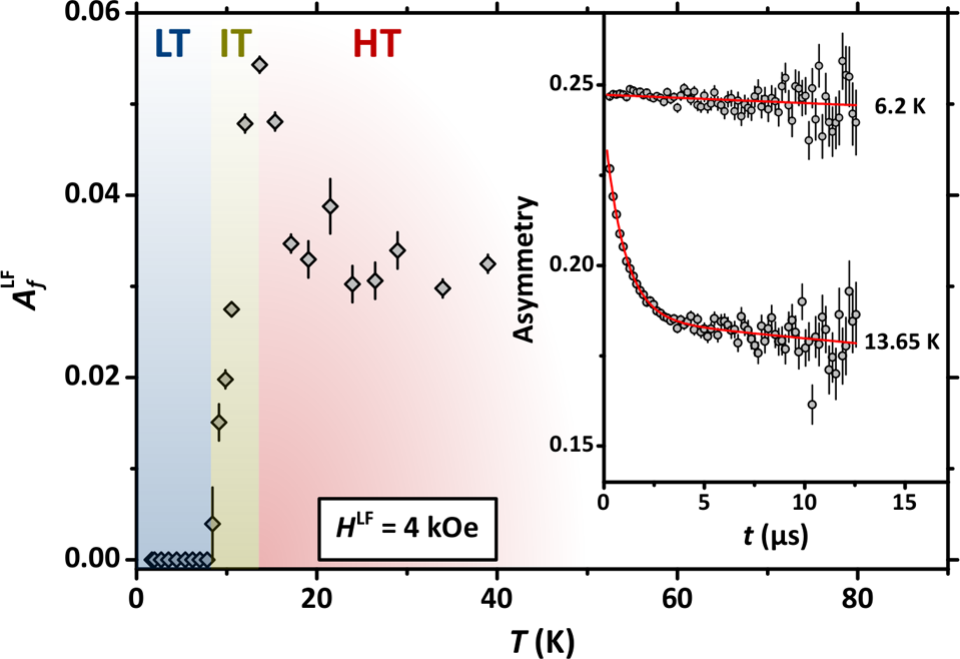
Main panel: temperature dependence of the amplitude of the initial exponentially decaying component for LF-μSR at *H*^LF^ = 4 kOe. Inset: representative results of LF-μSR at different temperature values and *H*^LF^ = 4 kOe. The continuous lines are best-fitting functions according to eq 2.

The central assumption in our analysis is that the different contributions in eq 1 and eq 2 are associated with signals from inequivalent implantation sites for the muons. We start from the discussion of the long-tail exponentially damped component on temperature, which is preserved qualitatively unmodified across the whole investigated temperature range. Indeed, we find *A*_*s*_^ZF^ ≃ 0.05–0.06 for the amplitude of this component at all the investigated temperatures. We report in Figure 3 the temperature dependence of the associated relaxation rate λ_*s*_^ZF^ as well as λ_*s*_^LF^ for the longitudinal field *H*^LF^ = 4 kOe. The observed trend can be interpreted as being due to dynamical influences on the muon depolarization via spin–lattice-like relaxation processes. In the fast-fluctuations regime, the relation
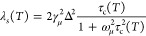
3holds,^23^ where τ_c_ is the correlation time for the dynamics being probed by the muons, Δ is the root-mean-square amplitude of the fluctuations of the local magnetic fields, and ω_μ_ = γ_μ_*H*^LF^ is the Larmor frequency for the muon. Assuming that
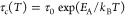
4where *k*_B_ is the Boltzmann constant, it is possible to quantify *E*_A_, i.e., the characteristic activation energy for the rotations, and the correlation time at infinite temperature τ_0_ by performing a simultaneous fit to the ZF and LF data for λ_*s*_(*T*) based on eq 3 and eq 4 for temperatures above *T* ∼ 10 K. The results of the fitting procedure are shown as dashed lines in the main panel of Figure 3. Based on our results, we estimate the remarkably low value *E*_A_ = 30 ± 2 cal mol^–1^, as well as τ_0_ = 3.5 ± 0.5 ns. We argue that the dynamical process probed by the muons is the rotation of the bicyclo-[1.1.1]-pentane rotors and, accordingly, both *E*_A_ and τ_0_ are characteristics of this rotary motion.

Some comments are in order at this stage. The muons contributing to the signal with amplitude *A*_*s*_ must be implanted in a site far enough from the rotor to form no entangled states with the nuclear moments of hydrogens but still close enough to probe via dipolar interactions the time-modulated magnetic fields generated by the moving rotors. Accordingly, a reasonable hypothesis is that these muons are localized on the stators. We also note that, as reported the Supporting Information (see Figure SI4), the recovery of the muon depolarization under the effect of an increasing longitudinal magnetic field shows that, after thermalizing in the sample, a major fraction of incoming muons form muonium, i.e., a bound μ^+^*e*^–^ state.^32^ This is ultimately justifying the low initial asymmetry value in ZF measurements.

It is also meaningful to compare our results from μSR with those collected by ^1^H NMR in the absence of implanted muons.^19^ The values for both *E*_A_ and τ_0_ estimated by muons are higher than the estimates made by means of the NMR spin–lattice relaxation rate; in fact, *E*_A_^NMR^ ≃ 6 cal mol^–1^ and τ_0_^NMR^ ≃ 4 ps.^19^ In this respect, it should be remarked that muons are not passive spectators in the system but are perturbing the rotors in a nontrivial way. In particular, the delicate combination of the different *C*_3_ and *C*_4_ symmetries of the rotors and of the stators, respectively, which is ultimately allowing the preservation of motions at cryogenic temperatures, is expected to be broken by the implantation of muons, which are adding charge in an asymmetric way. Accordingly, the detection of the onset of slower dynamical processes at temperatures higher than in the NMR measurements is reasonable. In spite of these arguments, muons still allow the estimate of an exceptionally low value for *E*_A_, confirming the easiness of rotation of the studied molecular rotors.

We now turn to the behavior of the other two components, *A*_*f*_1__^ZF^ and *A*_*f*_2__^ZF^, whose amplitudes sum up to ∼20–25% of the maximum instrumental asymmetry. In the LT regime, both these components give rise to characteristic oscillations in ZF, see Figure 2. Although we can use eq 1 at all of the temperature values, we notice that better fitting results are obtained for *T* ≲ 4 K by referring to the so-called H−μ state, see Figure SI6. In fact, in the absence of long-range ordered magnetism in the investigated material, well-defined oscillations in the ZF-μSR spectra are usually caused by the generation of quantum entangled states between the magnetic moments of the muons and the nuclei.^33−35^ Particularly relevant for our study is the H−μ state, where the entangled state is between the spin-1/2 nuclear spin of an hydrogen and the muon spin (*s*_μ_ = 1/2). As discussed in the Supporting Information, our data are consistent with the generation of two distinct H−μ states well within the LT regime. Based on the conventional static model (see eq SI11) we quantify precisely *r*_1_ = 1.46 ± 0.02 Å and *r*_2_ = 0.860 ± 0.015 Å for the characteristic muon–hydrogen distances in the two entangled states. We stress that the strong coupling with H nuclei identifies two muon thermalization sites directly onto the rotor unambiguously. Indeed, the other nuclear species in the investigated MOF are characterized by vanishing magnetic moments and hydrogens are only located as a crown on the central plane of the bicyclo-[1.1.1]-pentane rotating moieties (see Figure 1).

However, given the extremely low activation energy of ∼30 cal mol^–1^, the system still experiences rotary motion in the fast limit regime even at cryogenic temperatures, and the motional averaging should reduce the apparent frequency values accordingly. Therefore, the muon–hydrogen dipolar coupling must be averaged anisotropically because of the fast spinning of the muon,^36^ and the distances *r*_1_ and *r*_2_ estimated from a static H−μ model should be renormalized as well. In the assumption of an averaging by a factor 1/2 accounting for the muon rotating on a plane perpendicular to the rotor main axis,^37^ the renormalized distances would be *r*_1_′ ≃ 1.16 Å and *r*_2_′ ≃ 0.68 Å. This matches well with muons located between the hydrogens on the rotor in a collinear, though asymmetric, H−μ–H entangled state. Any attempt to make these arguments more quantitative should rely on a DFT modeling of the muon implantation site and of the possible distortions in the crystallographic structure induced by the muon implantation itself. This is beyond the current scope of this study. Also, the data within the IT region show evidence of a single oscillating component, but they cannot be described by the H−μ fitting function even after fixing one of the two oscillating amplitudes functions to zero. The more empirical fitting function reported in eq 1 gives excellent results instead, as shown in Figure 2, with *B*_1_ ≃ 12 G. Overall, this behavior may be due to a sudden change in the implantation sites upon increasing temperature, leading the muons to interact simultaneously with more hydrogens. The functional form for the aligned H−μ–H makes the fitting quality worse, suggesting that a more subtle numerical analysis is required by assuming that the muon and the protons are not aligned.^34^

It is remarkable that the oscillations in the ZF depolarization curves can be observed only in the LT and IT regimes. As mentioned above, the single oscillation observed in the IT region becomes progressively overdamped into an initial exponentially decaying component upon increasing temperature into the HT region (see Figure 2). As the overdamping is complete at around *T* ≃ 13 K, at this temperature we can associate a characteristic angular frequency of the fluctuations ω ∼ γ_μ_*B*_1_ ∼ 1 rad μs^–1^ – corresponding to the motional narrowing condition.^21^ This value is too low to be accounted for by the motion of the rotating moieties or by intrinsic degrees of freedom (e.g., vibrations and librations). Accordingly, we interpret this finding as the result of the onset of dynamical processes associated with the diffusive motion of those muons implanted on the rotors. In particular, the onset of muon diffusion at such low temperatures may be favored by the faster and faster motion of the rotating moieties. To the best of our knowledge, this is the first report of such unusual behavior for the evolution of H−μ coherent oscillations as a function of temperature.

A strong hint toward the dynamical origin of the initial depolarization in the HT regime arises from the behavior of the μSR signal under the effect of a magnetic field in LF geometry. We show selected depolarization curves for *H*^LF^ = 4 kOe in the inset of Figure 4, evidencing a marked dependence on temperature. In the LT regime, we observe an almost flat depolarization with full initial asymmetry; however, we detect a clear onset of dynamical processes leading to a fast-decaying component of the muon depolarization upon increasing temperature. In the main panel of Figure 4 we plot the temperature behavior of *A*_*f*_^LF^ showing unambiguously that dynamical processes set in upon increasing temperature within the IT region. We also notice that *A*_*f*_^LF^ shows a well-defined maximum at *T* ≃ 13.5 K and that its amplitude is suppressed in the HT regime, leading to a markedly nonmonotonic behavior. As discussed in the Supporting Information (Figure SI5), this loss of signal is not compensated by the amplitude of the long-tail component *A*_*s*_^LF^. We argue that additional processes set in within the HT region lead to a nondetectable initial depolarization because of the pulse-limited time-resolution at ISIS.

In conclusion, we used muon-spin spectroscopy at cryogenic temperatures to highlight the exceptional dynamical properties of molecular rotors bridging adjacent metal oxide nanoclusters, as engineered in a low-density metal–organic framework. Muon-spin spectroscopy provided different complementary viewpoints of the rapid rotor dynamics, even at very low temperatures. Indeed, muons are implanted in several privileged implantation sites, i.e., with one perceiving the rotor dynamics likely from the stator nodes, in close proximity to the rotor itself, and others on the fast-reorienting moiety. Thanks to these favorable observatories, a rich picture of the dynamics emerged, and we estimated an exceptionally low energy barrier ∼30 cal mol^–1^ for rotations. We also detected a peculiar temperature dependence of the coherent oscillations arising from those muons implanted on the rotors and possibly diffusing at temperatures *T* ≳ 13 K, implying a nontrivial effect of rotations on the implantation itself. This study stresses the utility of muon-spin spectroscopy to identify motions in organic solids and enlarges the perspectives for its application to rotors, motors, and molecular machines in the solid state.

## Supporting Information Available

Additional details on the sample synthesis and characterization as well as on muon spin spectroscopy and our μSR data (PDF)
