# Supplementary material for: Molecular Rotors in a Metal–Organic Framework: Muons on a Hyper-Fast Carousel
Source: Nano Lett. 2020 Sep 1;20(10):7613–8. doi: 10.1021/acs.nanolett.0c03140 (PMC8011913; doi:10.1021/acs.nanolett.0c03140)
Supplement: Supplementary file 1 — nl0c03140_si_001.pdf [file nl0c03140_si_001.pdf]

Molecular rotors in a metal organic framework: muons on a hyper-fast carousel. Supporting information

Giacomo Prando,[*,†] Jacopo Perego,[‡] Mattia Negroni,[‡] Mauro Riccò,[¶] Silvia Bracco,[‡] Angiolina Comotti,[‡] Piero Sozzani,[‡] and Pietro Carretta[†]

†*Department of Physics, University of Pavia, I-27100 Pavia, Italy*

‡*Department of Materials Science, University of Milano Bicocca, I-20125 Milano, Italy*

¶*Department of Mathematical, Physical and Information Sciences, University of Parma, I-43124 Parma, Italy*

E-mail: giacomo.prando@unipv.it

Synthesis and characterization of the Zn-MOF sample

Synthesis

Two mother solutions were prepared: bicyclo-[1.1.1]-pentane-1,3-dicarboxylic acid (936.5 mg; 6 mmol) dissolved in 60 mL of anhydrous DMF and zinc acetate dihydrate (3.951 g; 18 mmol) dissolved in 60 mL of anhydrous DMF. The solution containing the ligand was partitioned into 20 vials (3 mL each) and in each vial 3 mL of the salt solution was added dropwise under continuous stirring. A white powder started to precipitate during the addition process. When the addition was completed, each vial was stirred for 30 minutes and then left at room temperature for 16 hours. Each vial was centrifuged and the solvent was exchanged 3 times

with dry DMF (3×3 mL in each vial) and 3 times with dry dichloromethane (3×3 mL in each vial). The solvent was removed and the crystalline powder was activated under high vacuum for 12 hours at 130°C. All the analysis and experiments were performed under inert atmosphere. The reported procedure was repeated several times to synthesize gram-scale quantities of Zn-MOF.

Powder x-ray diffraction

Powder x-ray diffraction was performed at room temperature on a Rigaku Smartlab SE diffractometer operating with CuK_α radiation. The generator was set at 40 kV and 30 mA. The activated powder was placed on a zero-background silicon wafer sample holder and sealed under nitrogen atmosphere. The measurement was performed between 2°and 80° (2θ degrees) – see Fig. SI1. In order to eliminate the contribution to the diffraction pattern due to the Kapton window, a measurement with the empty sample holder was performed and the background signal subtracted from the raw data.

Nitrogen adsorption isotherm

Nitrogen adsorption data at 77 K were collected on a Micromeritics ASAP 2020 HD – see Fig. SI2. The surface area was calculated from the N_2 adsorption isotherm using the data in the pressure range between 0.015 and 0.1 p/p°, according to Langmuir models. Pore size distributions were calculated according to density functional theory.

Infrared spectroscopy

Infrared spectra were collected on a Jasco FT/IR 4100 equipped with an ATR PRO450-S module – see Fig. SI3. The sample was treated under vacuum before analysis to ensure the complete removal of adsorbed chemical species. Spectra were collected between 600 and 4000 cm^{-1} with a resolution of 2.0 cm^{-1}.

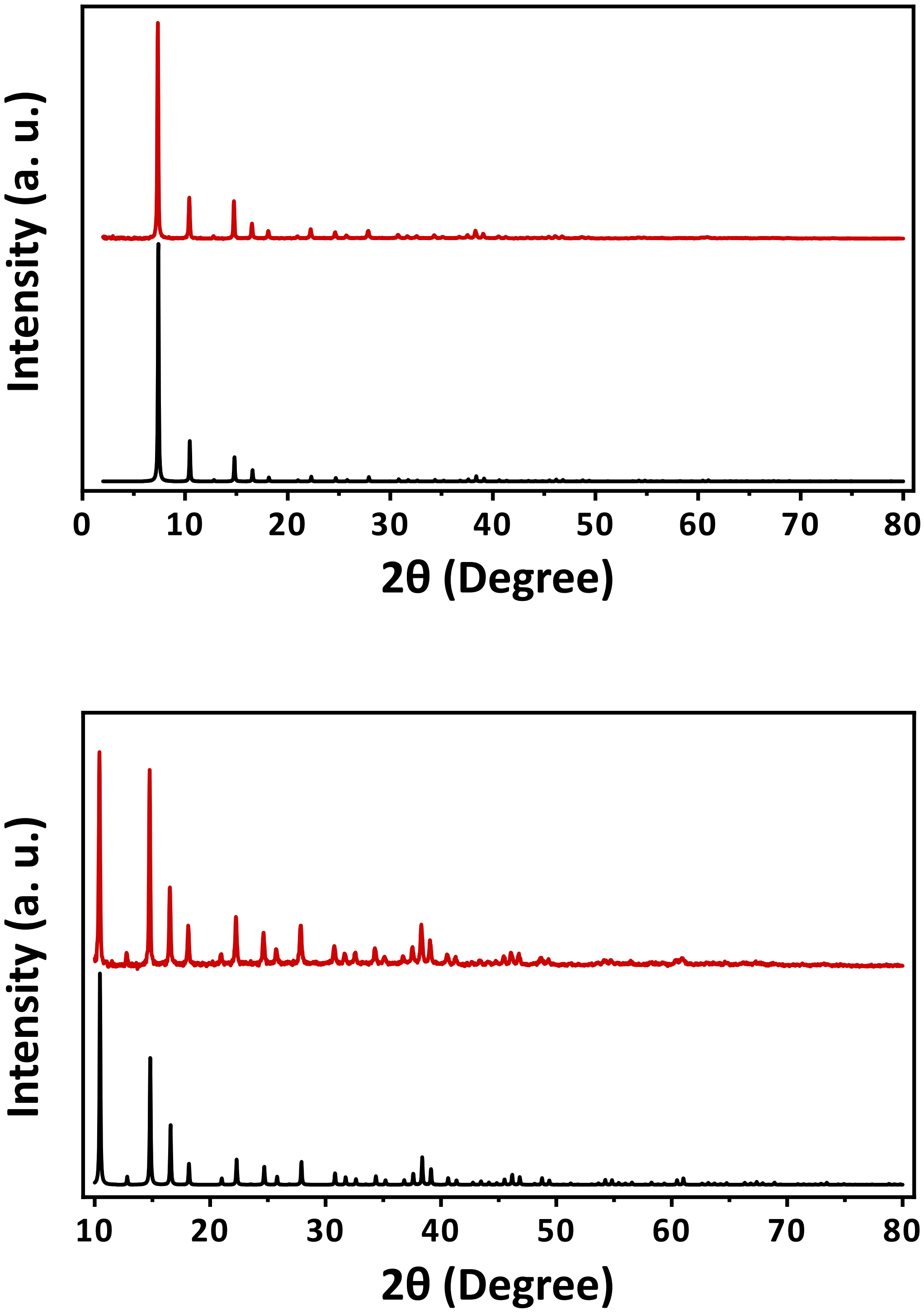

FIG. SI 1: Top panel: powder x-ray diffraction pattern of Zn-MOF collected from 2° to 80° (top, red) and calculated diffraction pattern from single crystal x-ray diffraction (bottom, black) (cubic crystal structure with the space group F-43c). Bottom panel: enlargement of powder x-ray diffraction pattern of Zn-MOF between 10° and 80° (top, red) and calculated diffraction pattern from single crystal x-ray diffraction (bottom, black).

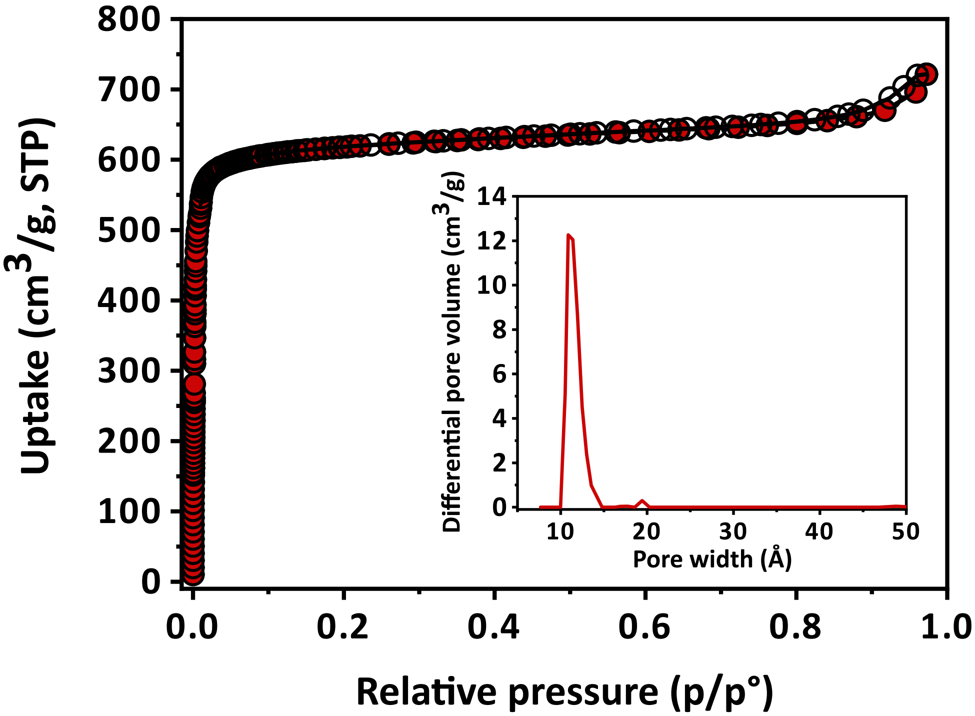

FIG. SI 2: Nitrogen adsorption isotherm (77 K) collected for the Zn-MOF sample. Inset: Pore size distribution calculated according to NLDFT method. Surface areas of 2680 and 2385 m^2/g were calculated according to Langmuir and BET models, in excellent agreement with the reported area.

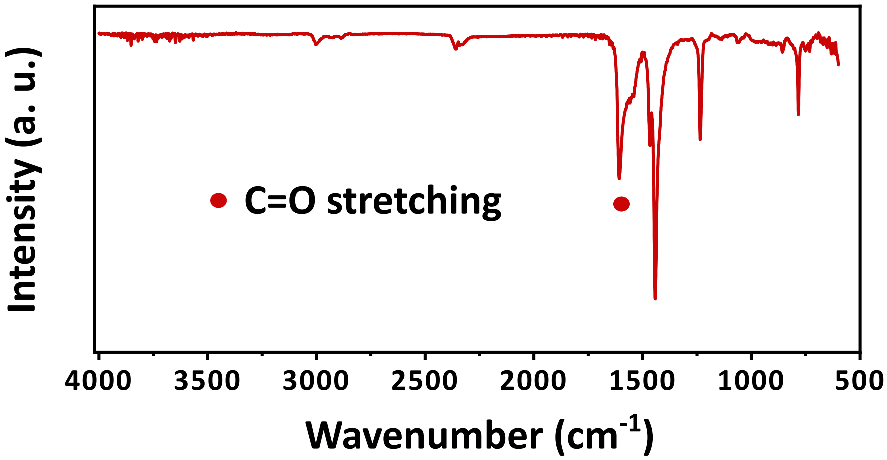

FIG. SI 3: Infrared spectra of the Zn-MOF sample. IR (ATR, cm^{-1}): 3002 (w), 1608 (vs), 1467 (s), 1442 (vs), 1235 (s), 783 (s). The C=O stretching band at 1608 cm^{-1} is shifted from 1684 cm^{-1} in the freed ligand, owing to the coordination with Zn ions.

Muon-spin spectroscopy

Generalities

In a muon-spin spectroscopy (μSR) experiment,[1–4] millions of spin-polarized positive muons μ^+ are implanted into the investigated sample. Once thermalized in the characteristic interstitial crystallographic position(s), the muon spins precess around the local magnetic field B_μ with angular frequency $\omega = \gamma_\mu B_\mu$, where $\gamma_\mu = 2\pi \times 13.554$ rad ms^{-1} G^{-1} is the gyromagnetic ratio for μ. After a mean lifetime ~ 2.2 μs, every muon decays into a positron and a neutrino/antineutrino couple and, due to the parity violation governing weak decays, the positron is emitted preferentially along the muon spin at the moment of the decay. Accordingly, based on a time- and space-resolved detection of positrons, it is possible to derive the evolution of the μ spin polarization between the moments of implantation and decay. The quantity of interest is the so-called time-dependent asymmetry function defined as

$$A(t) = \frac{N_B(t) - \alpha N_F(t)}{N_B(t) + \alpha N_F(t)}. \tag{SI1}$$

Here, the muon thermalization in the sample defines $t = 0$, $N_B(t)$ and $N_F(t)$ represent the positron counts at the time t in two detectors oppositely located with respect to the sample, and α is a calibration parameter accounting for non-equivalent conditions of the detectors. It can be shown that the spin autocorrelation function for the muon is defined as

$$G_T^{\mathrm{s}}(t) = \frac{A_T(t)}{A_T(0)} \tag{SI2}$$

where T accounts for the temperature value.

Experimental details

We performed μSR measurements on the EMU spectrometer of the ISIS pulsed muon source at the Rutherford Appleton Laboratories, UK. We sealed the sample into a Ti sample holder

with a 75-μm-thick kapton foil under inert atmosphere in order to prevent any contact with air and moisture at any stage before and during the experiment. Two additional 50-μm-thick silver foils were used as degraders to facilitate the implantation of the muons into the investigated sample. We investigated the temperature range 1.6 K $\leq T \leq$ 60 K using a ^{4}He-flow cryostat both in conditions of zero external magnetic field (ZF) and for applied magnetic fields $H^{\mathrm{LF}} \leq 4$ kOe in longitudinal geometry (LF), i.e., parallel to the muon spin at the moment of implantation.

Supplementary data

Here, we discuss additional μSR data on the investigated Zn-MOF. We report the two fitting functions for the ZF and LF geometries (Eqs. 1 and 2 in the main text, respectively)

$$A^{\mathrm{ZF}}(t) = \sum_{i=1}^{2} A_{f_i}^{\mathrm{ZF}} \cos\left(\gamma_\mu B_i t\right) e^{-\lambda_{f_i}^{\mathrm{ZF}} t} + A_s^{\mathrm{ZF}} e^{-\lambda_s^{\mathrm{ZF}} t} \tag{SI3}$$

and

$$A^{\mathrm{LF}}(t) = A_f^{\mathrm{LF}} e^{-\lambda_f^{\mathrm{LF}} t} + A_s^{\mathrm{LF}} e^{-\lambda_s^{\mathrm{LF}} t} \tag{SI4}$$

for convenience. The meaning of the symbols is thoroughly explained in the main text.

Longitudinal magnetic field scans. We performed measurements at selected temperature values as a function of the intensity of the longitudinal magnetic field H^{LF}. We performed a best-fitting procedure based on Eq. SI4 for all the investigated conditions. The fitting results for A_f^{LF} and A_s^{LF} are reported in the upper panels of Fig. SI4 for data taken at two different temperature values. The data have been corrected in order to take into account the weak dependence of the calibration parameter α in Eq. SI1 on the magnetic field.[5] The results at $T = 1.6$ K show clearly that the initial amplitude A_f^{LF} is completely quenched by the application of magnetic fields as low as $H^{\mathrm{LF}} \sim 30$ Oe, which is consistent with the static origin of the depolarization in the low-temperature regime. On the other hand, the results at $T = 19.1$ K show that the initial amplitude A_f^{LF} is not affected by H^{LF} up to the

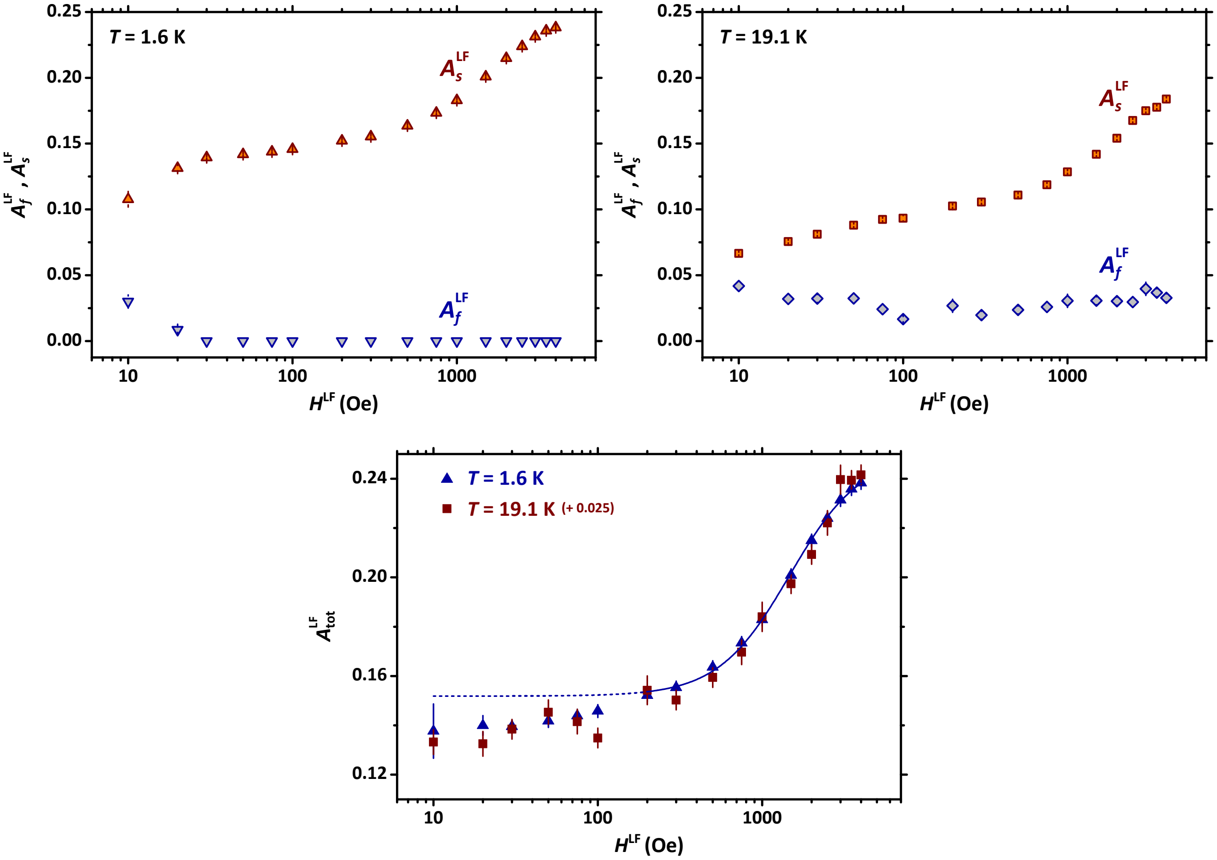

FIG. SI4: Top panels: longitudinal-field dependence of the initial asymmetry of the decaying components defined in Eq. SI4 for different temperature values. The data have been corrected to take into account the field dependence of α – see Eq. SI1. Bottom panel: longitudinal-field dependence of the total initial asymmetry defined in Eq. SI5. The data at taken at $T = 19.1$ K have been shifted vertically by a constant amount +0.025. The curve is derived after a best-fitting procedure to the experimental data taken at $T = 1.6$ K based on Eq. SI6. The line is continuous in the actual fitting range while the dashed section shows how the resulting curve behaves outside the fitting range.

maximum accessible value, confirming its dynamical nature in the high-temperature regime. These results are fully consistent with the data discussed in the main text.

The lower panel of Fig. SI4 shows the dependence of the total initial asymmetry

$$A_{\mathrm{tot}}^{\mathrm{LF}} = A_f^{\mathrm{LF}} + A_s^{\mathrm{LF}} \tag{SI5}$$

on H^{LF}. After shifting the data taken at $T = 19.1$ K by a constant value +0.025, all the results collapse onto a common trend. The need for this constant offset can be understood

based on the observation of a missing fraction in the temperature-dependent scans (see below in Fig. SI5).

The results in the lower panel of Fig. SI4 provide a definitive explanation for the low value for the total initial asymmetry in ZF. In particular, the observed recovery of $A^{\mathrm{LF}}_{\mathrm{tot}}$ upon increasing H^{LF} is the fingerprint of the formation of muonium – i.e., the bound state of a positive muon with an electron.[3,6] In the presence of muonium, the following expression is expected for the field dependence of the asymmetry

$$A_{\mu^+e^-}(H^{\mathrm{LF}}) = A_\infty \frac{\left(1-\frac{f}{2}\right)+\left(H^{\mathrm{LF}}/H_{\mathrm{hyp}}\right)^2}{1+\left(H^{\mathrm{LF}}/H_{\mathrm{hyp}}\right)^2}. \quad \text{(SI6)}$$

Here, A_∞ is the asymmetry value at high values of H^{LF} while the parameter f quantifies the fraction of incoming muons forming a bound state with an electron. Finally, the characteristic field H_{hyp} depends on the hyperfine interaction between the muon and the electron. In the simplified case of a free isotropic state in vacuum, one has

$$H_{\mathrm{hyp}} = \frac{D_{\mathrm{hyp}}}{\gamma_\mu + \gamma_e}, \quad \text{(SI7)}$$

where D_{hyp} quantifies the hyperfine coupling and γ's are the gyromagnetic ratios for the muon and the electron ($\gamma_\mu = 2\pi \times 13.554$ rad ms^{-1} G^{-1} and $\gamma_e = 2\pi \times 2.8024$ rad μs^{-1} G^{-1}). From the vacuum value $D_{\mathrm{hyp}} = 2\pi \times 4.4633$ rad ns^{-1}, the value $H_{\mathrm{hyp}} \simeq 1585$ G is expected as the result of a longitudinal magnetic field scan.[3]

We used Eq. SI6 as best-fitting function to our experimental data at $T = 1.6$ K shown in the lower panel of Fig. SI4. We performed the fit from 200 Oe to 4 kOe – in this field region, the best-fitting curve is plotted as continuous while outside the fitting range it is plotted as dashed. Based on our findings, we estimate $f \simeq 0.8$ for the fraction of incoming muons forming a bound state with the electrons and $H_{\mathrm{hyp}} \simeq 1470$ Oe (slightly lower values for H_{hyp} if compared to the expectations in vacuum are observed generally in condensed matter[6]). At the same time, the departure from Eq. SI6 for $H^{\mathrm{LF}} \lesssim 200$ Oe can

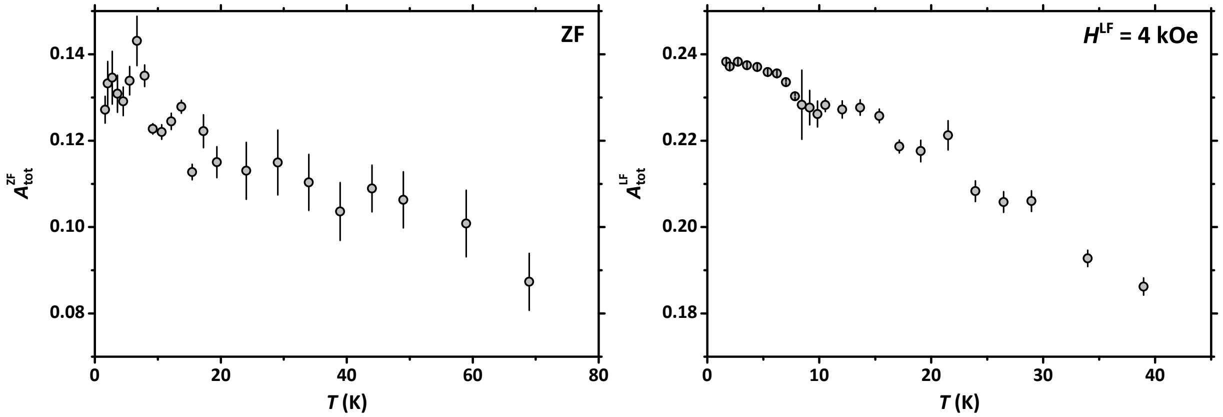

FIG. SI5: Temperature dependence of the total initial asymmetry defined in Eq. SI8 for ZF conditions (left-hand panel) and in Eq. SI5 in LF geometry (right-hand panel). Notice the varying ranges covered by the axes in the different panels.

be an indication of an anisotropic contribution to the hyperfine coupling.[6] All these aspects deserve further investigation under different experimental conditions in order to achieve a better understanding of the muonium state in the investigated material.

Temperature scans. In Fig. SI5, we report the temperature dependence of the total asymmetry $A_{\mathrm{tot}}^{\mathrm{ZF}}$, defined as follows

$$A_{\mathrm{tot}}^{\mathrm{ZF}} = \sum_{i=1}^{2} A_{f_i}^{\mathrm{ZF}} + A_s^{\mathrm{ZF}}, \tag{SI8}$$

as well as the temperature dependence of the total asymmetry $A_{\mathrm{tot}}^{\mathrm{LF}}$ defined in Eq. SI5. From the results, it is evident that an asymmetry value ~ 0.04 is lost upon increasing temperature from the LT region to the HT regime regardless of the applied magnetic field. We interpret this as the development of a fast-decaying component not detectable because of the pulsed nature of the muons beam at ISIS. Further investigations are needed in order to clarify the origin of this missing fraction with better time-resolution and, possibly, at different conditions for the external magnetic field.

Sum of two entangled H–μ states – Fitting function

Within the model for an entangled H–μ state, the following expression is expected for the time-evolution of the ZF-μSR signal[4,7]

$$A^{\mathrm{H}\mu}(t) = \frac{1}{6}\left[1 + \sum_{k=1}^{3} a_k \cos\left(\frac{k}{2}\gamma_\mu B_{\mathrm{d}} t\right)\right] e^{-\lambda_f^{\mathrm{ZF}} t} \tag{SI9}$$

where $a_1 = a_3 = 2$, $a_2 = 1$ and

$$B_{\mathrm{d}} = \frac{\hbar \gamma_{\mathrm{H}}}{r^3}. \tag{SI10}$$

As shown by the fitting curve for the data at $T = 2.05$ K in Fig. 2 (main text), it is possible to reproduce our ZF-μSR measurements well within the LT region assuming the generation of two distinct H–μ entangled states. According, for the overall fitting function we write

$$A(t) = \sum_{i=1}^{2} A_i^{\mathrm{H}\mu}(t) + A_s^{\mathrm{ZF}} e^{-\lambda_s^{\mathrm{ZF}} t}. \tag{SI11}$$

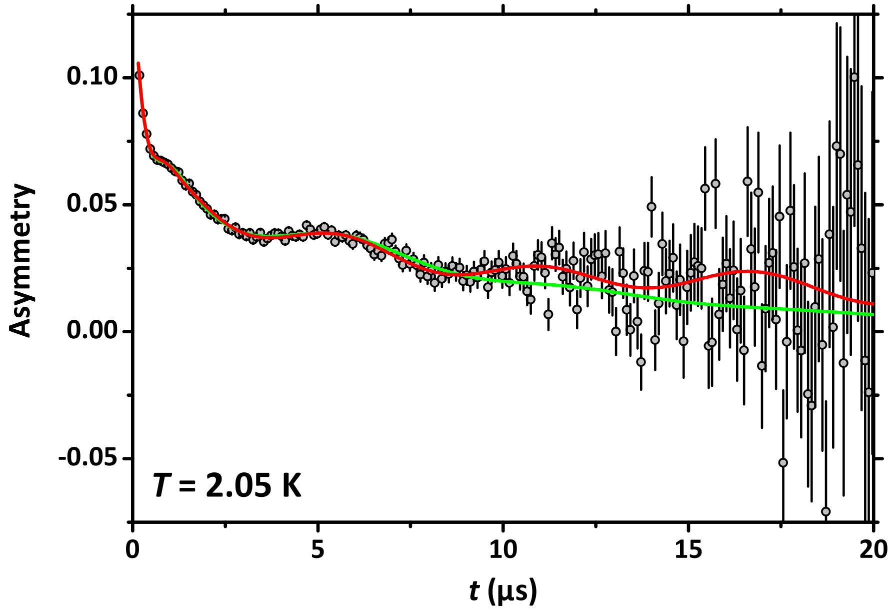

FIG. SI6: Results of ZF-μSR at 2.05 K, already shown in Fig. 2 in the main text. The red continuous line is a best-fitting function according to the H–μ model – see Eq. SI11. The green continuous line is a best-fitting function according to Eq. 1 in the main text.

This is shown further in Fig. SI6, where we compare the results of a fitting procedure based on Eq. SI11 and on Eq. 1 from the main text (red and green curves, respectively). The model based on the sum of signals from two entangled H–μ states leads to a better agreement with the experimental data for $t \gtrsim 8$ μs.

References

(1) Cox, S. F. J. Implanted muon studies in condensed matter science. *Journ. Phys. C* **1987**, *20*, 3187.

(2) Blundell, S. J. Spin-polarized muons in condensed matter physics. *Contemp. Phys.* **1999**, *40*, 175.

(3) Blundell, S. J. Muon-Spin Rotation Studies of Electronic Properties of Molecular Conductors and Superconductors. *Chem. Rev.* **2004**, *104*, 5717.

(4) Yaouanc, A.; Dalmas de Réotier, P. *Muon Spin Rotation, Relaxation, and Resonance: Applications to Condensed Matter*; Oxford University Press, 2011.

(5) EMU User Guide (February 2020). 2020; `www.isis.stfc.ac.uk/Pages/emu\_manual\_20200213.pdf`.

(6) Pratt, F. L. Repolarization of anisotropic muonium in orientationally disordered solids. *Phil. Mag. Lett.* **1997**, *75*, 371.

(7) Lancaster, T.; Blundell, S. J.; Baker, P. J.; Brooks, M. L.; Hayes, W.; Pratt, F. L.; Manson, J. L.; Conner, M. M.; Schlueter, J. A. Muon-Fluorine Entangled States in Molecular Magnets. *Phys. Rev. Lett.* **2007**, *99*, 267601.
